# Disentangling the GRADE Domains of Inconsistency and Imprecision: An Approach Based on a Meta‐Research Study

**DOI:** 10.1002/cesm.70102

**Published:** 2026-08-31

**Authors:** Bernardo Sousa‐Pinto, Antonio Bognanni, Manuel Marques‐Cruz, Ignacio Neumann, Rafael José Vieira, Sara Gil‐Mata, Elie Akl, Joerg J. Meerpohl, Holger J. Schünemann, Waldemar Siemens

**Affiliations:** ^1^ MEDCIDS—Department of Community Medicine, Information and Health Decision Sciences, Faculty of Medicine University of Porto Porto Portugal; ^2^ CINTESIS@RISE—Health Research Network, Faculty of Medicine, MEDCIDS University of Porto Porto Portugal; ^3^ IRCCS Humanitas Research Hospital & Department of Biomedical Sciences, Clinical Epidemiology and Research Center (CERC) Humanitas University Milan Italy; ^4^ School of Medicine Universidad San Sebastián Santiago Chile; ^5^ Department of Internal Medicine American University of Beirut Beirut Lebanon; ^6^ Medical Center—University of Freiburg/Medical Faculty—University of Freiburg, Institute for Evidence in Medicine Freiburg Germany; ^7^ Cochrane Germany Cochrane Germany Foundation Freiburg Germany; ^8^ Fraunhofer Institute for Translational Medicine and Pharmacology ITMP Allergology and Immunology Berlin Germany

**Keywords:** decision thresholds, GRADE, imprecision, inconsistency, meta‐research

## Abstract

**Introduction:**

The assessment of the certainty of evidence using GRADE requires evaluating inconsistency and imprecision. These domains are difficult to judge in a fully independent way. We performed a meta‐research study to propose an approach to disentangle inconsistency and imprecision based on decision thresholds.

**Methods:**

We evaluated systematic reviews published in the Cochrane Database of Systematic Reviews. We included all meta‐analyses published in these reviews (i) with at least five primary studies, (ii) which did not correspond to subgroup analyses, and (iii) which had provided their results as continuous outcomes or as dichotomous outcomes with previously established decision thresholds. We recalculated each included meta‐analysis using the fixed‐ and the random‐effects models, retrieving metrics potentially expressing inconsistency or imprecision. This includes inconsistency indices, variables related to the confidence intervals (CI) of the meta‐analytical estimate and prediction intervals. Based on these variables, we performed factor analysis, hierarchical cluster analysis, and decision tree analysis.

**Results:**

We assessed 8703 meta‐analyses. The factor analysis resulted in the identification of three factors: (i) one factor associated with inconsistency metrics, (ii) one factor associated with imprecision metrics, and (iii) one factor of metrics not clearly related to inconsistency or imprecision. Taken together, the analysis suggests an approach based on the number of decision thresholds crossed by the CI of the fixed‐ and random‐effects meta‐analytical estimates: the number of thresholds crossed by the CI of the fixed‐effect model informs about imprecision, while the difference in the number of thresholds crossed by the CIs of the random‐ versus the fixed‐effect models reflects inconsistency. Prediction intervals and inconsistency indices, such as the *I* ^2^, can help detect cases in which inconsistency may be underestimated.

**Conclusion:**

Our results may help those assessing the certainty in evidence to independently rate inconsistency and imprecision.

## 1 Introduction

In the GRADE approach to rate the certainty in a body of evidence, inconsistency relates to the variability of effect sizes in primary study results [1], while imprecision ratings reflect uncertainty based on random error [2]. However, despite being two different sources of uncertainty, these two domains are closely related to each other, and difficult to assess in a fully independent way. In fact, both domain ratings are influenced by if the quantitative estimate of interest crosses decision thresholds (DTs) [1, 3, 4]. DTs are “reference values” that define whether effect sizes are trivial (or none), small, moderate or large [5, 6]. The definition of DTs is a crucial aspect for the assessment of certainty of evidence according to GRADE guidance, and judging the different domains should take into account DTs (https://book.gradepro.org/).

**Figure 1:**
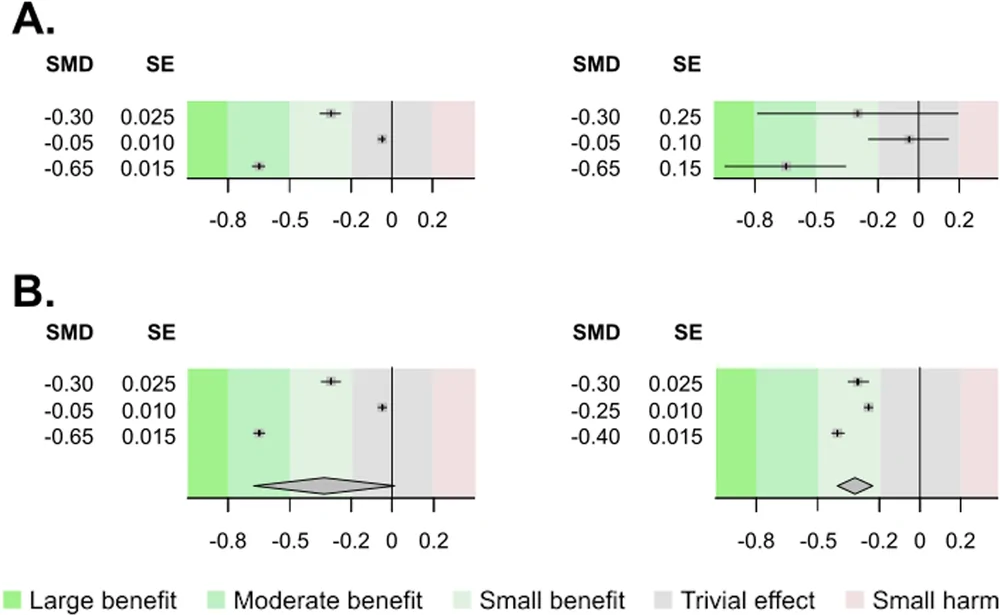
Graphical representation of the challenges of independently rating inconsistency and imprecision. A: Rating for inconsistency requires an assessing if the point estimates of primary studies fall in the same certainty range. In the scenario on the left they are in different effect size ranges (suggesting serious inconsistency); in the scenario on right (lower precision, but the same point estimates for the effect sizes of primary studies), the confidence intervals for the different primary studies consider the possibility that the real effect size is associated with a small benefit. B: Rating imprecision requires assessing the number of decision thresholds crossed by the meta‐analytical estimate. In the scenario on the left, the meta‐analytical estimate crosses two decision thresholds (suggesting serious imprecision); in the scenario on the right (same precision, but lower inconsistency between effect sizes of primary studies), the meta‐analytical estimate does not cross any decision threshold. SE, Standard‐error; SMD, Standardised mean difference.

The levels of rating down for imprecision reflect the number of DTs crossed by the confidence interval (CI) of the meta‐analytical estimate [3]. However, in the random‐effects model of a meta‐analysis, the width of the CI of the meta‐analytical estimate reflects not only within‐study variability (i.e., the imprecision of primary studies), but also between‐study variability [7] (related to inconsistency). That is, the higher the differences in the effect sizes of primary studies, the wider the CI of the random‐effects meta‐analytical estimate (and, potentially, the higher the number of DTs crossed by the meta‐analytical estimate) (Figure 1).

GRADE explicitly cautions against overpenalizing a body of evidence for both imprecision and inconsistency [3]. However, methods are needed that allow to assess these domains separately. In a meta‐analysis, measures commonly used to assess statistical heterogeneity (e.g., *I* ^*2*^ value or *Q* Cochran test *p*‐value) [7, 8] may help disentangling inconsistency from imprecision. However, these methods have some limitations, including (i) the fact that they may also be influenced by imprecision (e.g., the *I* ^*2*^ value may overestimate heterogeneity in meta‐analysis of studies with high precision or underestimate it when dealing with low precision studies [Box 1] [7]), (ii) the challenges of establishing a correspondence between the values of these measures and the number of levels to downgrade when judging inconsistency, and (iii) the fact that they do not take into account DTs. Prediction intervals (PI) can also inform about inconsistency [11]. However, a recent meta‐research study suggested that establishing a direct relation between the number of DTs crossed by PIs and the number of levels to downgrade for inconsistency may result in over‐penalising judgements. Even in meta‐analyses with ≥5 studies more than 60% of the meta‐analytical PIs crossed at least three DTs (Siemens et al. Under review). Another approach for disentangling inconsistency and imprecision involves comparing the number of DTs crossed by the CIs of the random and the fixed effects meta‐analytical estimates [4]. However, this approach has not been empirically tested.

Box 1 **Key concepts for the assessment of inconsistency and imprecision.**

**Meta‐analytical models:**

- **Fixed‐effects model:** Meta‐analytical model that assumes that all effect estimates are estimating the same underlying intervention effect, with differences between effect size measures of primary studies reflecting within‐study variability (random error) [7]. Under the fixed‐effects model, the effect sizes of primary studies are weighted based on their precision. Therefore, the width of the confidence interval of the meta‐analytical estimate primarily reflects the precision of the primary studies. That is, if the fixed effects model is applied to a meta‐analysis of inconsistent primary studies (something which is not recommended!), that would not affect the width of the confidence interval of the meta‐analytical estimate.
- **Random‐effects model:** Meta‐analytical model that assumes that the different primary studies are estimating different intervention effects, so that differences between effect size measures of primary studies reflect both within‐study and across‐study variability (i.e., differences that cannot be explained by random error alone) [7]. Under the random‐effects model, the effect sizes of primary studies are weighted based on their precision and on across‐study variability. Therefore, the width of the confidence interval of the meta‐analytical estimate reflects both the precision of the primary studies and the across‐studies variability. In this context, both inconsistency and imprecision affect the width of the confidence interval of the meta‐analytical estimate.

**Measures commonly used to assess heterogeneity:**

- **Cochran' *Q* test:** Hypothesis test based on the chi‐square statistic. It yields a *p*‐value enabling to reject or not the null hypothesis of homogeneity between primary studies’ effect size measures [7]. Its main limitations include the fact that this test (i) has low power in meta‐analyses of few primary studies and/or studies with small sample size, but is overpowered to detect small amounts of heterogeneity in meta‐analyses with a large number of primary studies, and (ii) does not take into account decision thresholds.
- ***I*** ^**2**^ **index:** Statistic frequently used to measure the relative extent of heterogeneity stemming from between‐study variability in a meta‐analysis, expressed on a scale of 0%–100% (higher values are suggestive or a higher amount of differences between studies) [7]. Its main limitations include the fact that this index (i) tends to yield biased results when used in small meta‐analyses and to overestimate heterogeneity in meta‐analysis of primary studies with precise estimates, and (ii) does not take into account decision thresholds [3, 8, 9].
- **Across‐studies inconsistency index:** Statistic used to measure the relative extent of heterogeneity, considering decision thresholds [10]. In particular, it assesses how inconsistent are the effect sizes of primary studies (in their distribution across information sizes) when compared among each other.
- **Decision inconsistency index:** Statistic used to measure the relative extent of heterogeneity, considering decision thresholds [10]. In particular, it assesses the spread of posterior samples of primary studies across information sizes defined by decision thresholds. It appears to be influenced not only by inconsistency but also by imprecision of the effect size measures.

Therefore, this GRADE‐related paper has two aims. The first aim is to test if the comparison between the number of thresholds crossed by the CIs of the random‐effects and the fixed‐effect model meta‐analytical estimates supports judgements about the number of levels to downgrade for imprecision and inconsistency. The second aim is to explore if inconsistency indices (such as the *I* ^2^) and PIs add to the differentiation of inconsistency and imprecision. To achieve these aims, we performed an empirical meta‐research assessment of systematic reviews published in the Cochrane Database of Systematic Reviews (CDSR).

## 2 Methods

### 2.1 Overview

**Figure 2:**
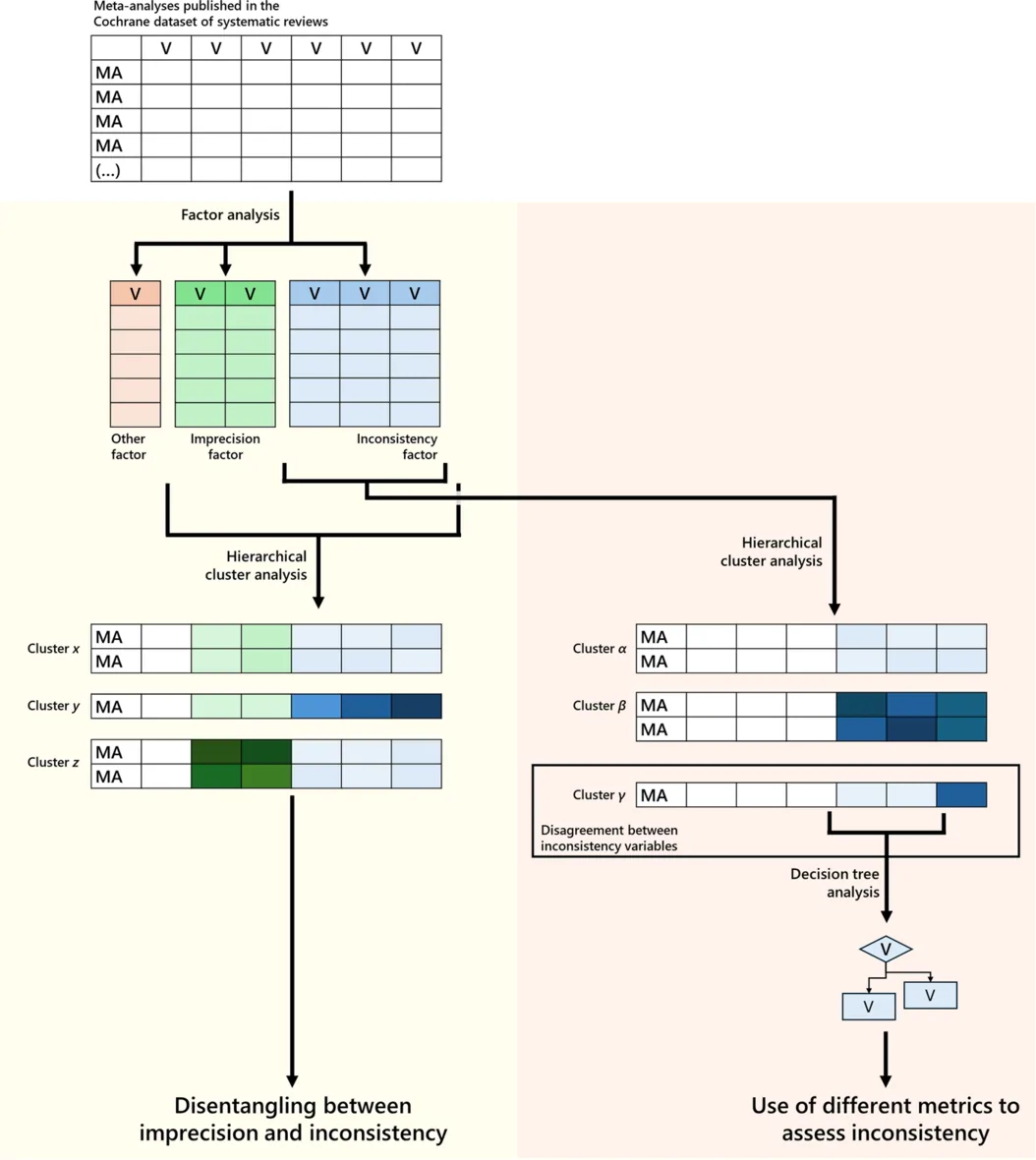
Diagrammatic representation of the study design. MA, meta‐analysis; V, variable.

In a set of systematic reviews published in CDSR, we computed a set of metrics that have been proposed to assess inconsistency or imprecision. To evaluate how to disentangle inconsistency and imprecision, we performed a factor analysis to assess (i) if we would be able to identify sets of metrics that were more closely associated with each other, and (ii) if the difference in the number of DTs crossed by the CIs of the random‐ and the fixed‐effect model estimates (∆DT) would be closely associated with other inconsistency measures. We then performed hierarchical clustering approaches based on imprecision‐ or inconsistency‐related variables to assess the robustness of the factor analysis. Subsequently, to evaluate the need of using different metrics to evaluate inconsistency, we performed a second hierarchical clustering analysis (based on inconsistency‐related variables only) followed by a decision tree analysis. A diagrammatic representation of the study design is available in Figure 2. This article follows the guidelines for reporting meta‐epidemiological methodology research proposed by Murad and Wang [12].

### 2.2 Data Analysis

A full description of the methodology of this study is provided in Supplementary Methods. In brief, in February 2024, we retrieved all systematic reviews published in the CDSR, with a valid Digital Object Identifier (DOI) and available meta‐analytical data. From these, we assessed all meta‐analyses (i) with at least five primary studies, (ii) that did not correspond to subgroup analyses, and that had provided their results (iii.a) as continuous outcomes allowing for computing meta‐analytical results as standardised mean differences (SMD), or (iii.b) as dichotomous outcomes for which DTs have been established in previous health guidelines (Table S1). We excluded diagnostic test accuracy and network meta‐analyses.

We recalculated each included meta‐analysis using the fixed‐effects model and the random‐effects model (Box 1). For continuous outcome variables, we performed meta‐analysis of SMD; for categorical outcome variables, we performed meta‐analysis of risk ratios (RR), odds ratios (OR) or risk differences (RD).

From each meta‐analysis, we retrieved metrics related to imprecision and inconsistency (Supplementary Methods S1). We performed a factor analysis to identify factors of metrics. Factor analysis is an approach that allows to identify variables that are strongly interrelated/correlated with each other; it assumes these interrelationships to be potentially explainable by underlying variables called “factors” [13]. Therefore, our factor analysis identifies sets of metrics more closely associated with each other (and potentially preferably pointing to inconsistency or imprecision). The list of variables used for factor analysis is available in Supplementary Methods and their main concepts are available in Box 1.

In order to assess the robustness of the factor analysis results, we applied hierarchical clustering methods based on variables more closely related to inconsistency and imprecision. In the dataset of meta‐analyses published in the CDSR, we obtained a representative example of the different clusters to discuss practical judgements for inconsistency and imprecision.

Finally, to assess whether different inconsistency measures provide complementary information, we performed a second hierarchical clustering. This clustering was based on inconsistency variables; that is, the metrics identified by the factorial analysis as informing about inconsistency. This hierarchical cluster analysis was followed by a decision tree to identify rules that would complement the sole use of the ∆DT to evaluate inconsistency (Figure 2).

We performed all analysis using the software R (version 4.5.0.). Generative AI was not used in any task of this manuscript.

## 3 Results

### 3.1 Meta‐Research Study

Out of the 132,197 meta‐analyses published in systematic reviews of the CDSR with available data and involving more than one primary study, 8703 (6.6%) met the eligibility criteria and were assessed (Figure S1). The median number of included primary studies was of 8 (percentiles 25–75 = 6–13), while the median number of assessed participants was of 1069 (percentiles 25–75 = 541–2449). A description of the included meta‐analyses is available in Table 1.

**Table 1:** Characteristics of the included meta‐analyses.

Variable	Included meta‐analyses (*N* = 8703)
* N* included primary studies – median (P25‐P75)	8 (6–13)
* N* participants—median (P25‐P75)	1069 (541–2449)
* I* ^2^ (%)—median (P25‐P75)	56.2 (12.6–83.4)
Q‐Cochran test *p*‐value—median (P25‐P75)	0.686 (0.186–1.000)
DI (%)—median (P25‐P75)	35.6 (17.1–51.5)
ASI (%)—median (P25‐P75)	28.8 (5.7–43.2)
*N* decision thresholds crossed by the CI of the FE meta‐analytical estimates—*N* (%)	
0	2769 (31.8)
1	4943 (56.8)
2	847 (9.7)
≥ 3	144 (1.7)
*N* decision thresholds crossed by the CI of the RE meta‐analytical estimates—*N* (%)	
0	1393 (16.1)
1	3500 (40.2)
2	2387 (27.4)
≥ 3	1423 (16.4)
Ratio of widths of RE/FE meta‐analytical estimate CIs—median (P25‐P75)	1.7 (1.1–2.7)
*N* decision thresholds crossed by the prediction interval—*N* (%)	
0	622 (7.1)
1	1315 (15.1)
2	1148 (13.2)
≥ 3	5618 (64.6)
EPSS – median (P25‐P75)	41.5 (20.8–58.7)

Abbreviations: ASI, Across‐studies inconsistency index; CI, Confidence interval; DI, Decision inconsistency index; EPSS, Expected proportion of studies summary; FE, Fixed‐effects; P, percentile; RE, Random‐effects.

#### 3.1.1 Disentangling Between Imprecision and Inconsistency

##### 3.1.1.1 Factor Analysis

The factor analysis resulted in the identification of three factors, with the proportion of variance explained by each factor being of 0.45 (factor 1), 0.17 (factor 2) and 0.11 (factor 3). The Bartlett's test *p*‐value was < 0.001, suggesting adequacy of factor analysis.

**Figure 3:**
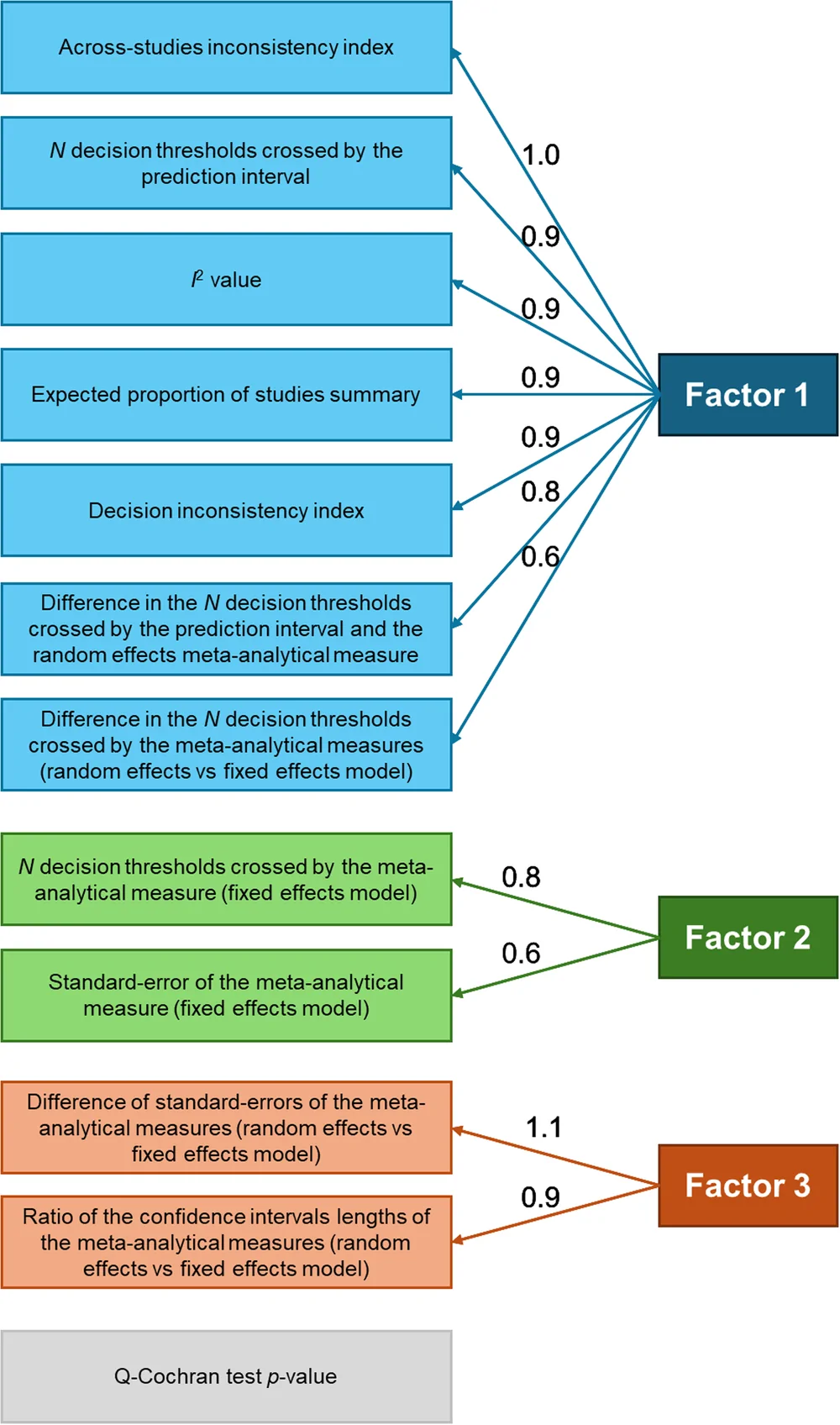
Diagram of the factor analysis identifying the loadings of the different variables associated with each factor.

The variables associated with each factor are displayed on Figure 3. In brief, (i) factor 1 was particularly associated with inconsistency, comprising seven variables, including ∆DT, inconsistency indices and the number of DT crossed by PI; (ii) factor 2 was particularly associated with imprecision, comprising two variables related to results under fixed effects model; and (iii) factor 3 (two variables) was not clearly related to either inconsistency or imprecision.

The Q‐Cochran test *p*‐value was not associated with any of the aforementioned factors (absolute value of the factor loading < 0.2 for the three factors).

##### 3.1.1.2 Hierarchical Cluster Analysis (Variables From Factor 1 and Factor 2)

We then applied hierarchical cluster analysis to cluster meta‐analyses according to their inconsistency and imprecision (variables that were part of factors 1 and 2 in factor analysis). The gap statistic pointed to an ideal number of seven clusters; however, since one of the generated clusters encompassed less than 10 meta‐analyses, we merged it together with the closest cluster, resulting in a total of six clusters. A quantitative description of each cluster is provided in Table 2, while an interpretative description with examples is provided in Box 2.

**Table 2:** Results of the hierarchical clustering approach based on imprecision and inconsistency metrics (*N* = 8703).

	Cluster I	Cluster II	Cluster III	Cluster IV	Cluster V	Cluster VI
*N* (%) meta‐analyses	289 (3.3)	93 (1.1)	4182 (48.1)	75 (0.9)	3894 (44.7)	170 (2.0)
**Variables that were part of factor 1 in factor analysis**						
* I* ^2^ – median (IQR)	0 (0)	0 (4.6)	23.4 (48.7)	0 (20.8)	80.1 (25.8)	97.0 (5.3)
DI—median (IQR)	20.9 (7.8)	35.3 (10.2)	17.1 (18.7)	55.1 (21.8)	51.6 (13.1)	64.1 (16.5)
ASI—median (IQR)	0.9 (0.5)	2.1 (3.2)	10.9 (23.1)	2.5 (17.9)	42.8 (13.2)	55.4 (14.8)
Difference of thresholds crossed by the CI of RE and FE meta‐analytical estimates—*N* (%)						
0	281 (97.2)	79 (84.9)	3394 (81.2)	47 (62.7)	909 (23.3)	0
1	8 (2.8)	11 (11.8)	723 (17.3)	23 (30.7)	1760 (45.2)	0
2	0	3 (3.2)	65 (1.6)	5 (6.7)	1007 (25.9)	2 (1.2)
≥3	0	0	0	0	218 (5.6)	168 (98.8)
*N* decision thresholds crossed by PI—*N* (%)						
0	28 (9.7)	2 (2.2)	589 (14.1)	2 (2.7)	0	1 (0.6)
1	33 (11.4)	0	1282 (30.7)	0	0	0
2	172 (59.5)	6 (6.5)	970 (23.2)	0	0	0
3	56 (19.4)	42 (45.2)	876 (20.9)	14 (18.7)	213 (5.5)	0
4	0	20 (21.5)	263 (6.3)	11 (14.7)	884 (22.7)	0
5	0	14 (15.1)	44 (1.1)	20 (26.7)	1016 (26.1)	0
6	0	9 (9.7)	158 (3.8)	28 (37.3)	1781 (45.7)	169 (99.4)
Difference of thresholds crossed by PI and the CI of the RE meta‐analytical estimate—*N* (%)						
0	191 (66.1)	19 (20.4)	1659 (39.7)	37 (49.3)	2 (0.1)	62 (36.5)
1	70 (24.2)	60 (64.5)	1064 (25.4)	29 (38.7)	113 (2.9)	55 (32.4)
2	27 (9.3)	12 (12.9)	1034 (24.7)	9 (12.0)	905 (23.2)	53 (31.2)
3	1 (0.3)	2 (2.2)	205 (4.9)	0	1711 (43.9)	0
4	0	0	91 (2.2)	0	1014 (26.0)	0
5	0	0	61 (1.5)	0	135 (3.5)	0
6	0	0	68 (1.6)	0	14 (0.4)	0
EPSS—median (IQR)	27.0 (7.7)	45.1 (16.9)	20.6 (20.6)	56.9 (22.9)	59.6 (15.3)	52.1 (25.1)
**Variables that were part of factor 2 in factor analysis**						
Standard‐error of the FE meta‐analytical estimate—median (IQR)	0.14 (0.03)	0.22 (0.09)	0.06 (0.05)	0.10 (0.07)	0.07 (0.04)	0.10 (0.05)
*N* decision thresholds crossed by FE meta‐analytical estimate—*N* (%)						
0	50 (17.3)	0	1964 (47.0)	2 (2.7)	740 (19.0)	13 (7.6)
1	62 (21.5)	2 (2.2)	2039 (48.8)	0	2717 (69.8)	123 (72.4)
2	177 (61.2)	50 (53.8)	179 (4.3)	0	412 (10.6)	29 (17.1)
≥ 3	0	41 (44.1)	0	73 (97.3)	25 (0.6)	5 (2.9)
**Variables that were part of factor 3 in factor analysis**						
Ratio of widths of RE/FE meta‐analytical estimate—median (IQR)	1.00 (0.01)	1.02 (0.06)	1.21 (0.61)	1.01 (0.36)	2.44 (1.72)	6.12 (6.10)
Difference of standard‐errors of RE and FE meta‐analytical estimates—median (IQR)	0 (0.01)	0.01 (0.02)	0.01 (0.03)	0 (0.02)	0.09 (0.10)	0.53 (0.46)
**Variables with no associated factor in the factor analysis**						
Significant Q‐Cochran *p*‐value—*N* (%)	15 (5.2)	4 (4.3)	587 (14.0)	7 (9.3)	1003 (25.8)	50 (29.4)

*Note:* Green indicates low values in inconsistency or imprecision metrics, yellow indicates low‐intermediate values, orange indicates high‐intermediate values, and red indicates high values.

Abbreviations: ASI, Across‐studies inconsistency index; DI, Decision inconsistency index; EPSS, Expected proportion of studies summary; FE, Fixed effects; IQR, Interquartile range; RE, Random effects.

Box 2 **Examples for some of the assessed clusters on the application of approaches to disentangle inconsistency from imprecision.**

We provide four examples, including one example of a meta‐analysis of cluster I (and II), one of cluster III, one of cluster IV, and one of cluster V (and VI). The horizontal axis of the plots displays either standardised mean differences (continuous outcomes) or absolute risk differences (dichotomous outcomes). The background colours indicate the following:

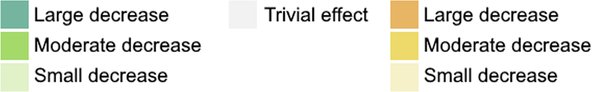

**Example for cluster I (and II)**

Fraison et al. have performed a systematic review comparing metformin *versus* the combined oral contraceptive pill in women with polycystic ovary syndrome [14]. One of the performed meta‐analysis had the body mass index as its outcome. The meta‐analysis comprised 6 primary studies, including a total of 199 participants. The random‐effects SMD was −0.45 (95%CI = −0.74; −0.17) [*I* ^2^ = 10.4%; DI = 22.0%; ASI = 0.7%]. Considering the usual thresholds for SMDs, the random‐effects meta‐analytical estimate crosses two decision thresholds (moderate/small decrease and trivial/small decrease). The fixed effects SMD would be −0.45 (95%CI = −0.74; −0.17), with two decision thresholds being crossed.

The fact that both the CI of the fixed effects and of the random effects meta‐analytical estimate cross two decision thresholds indicates that there may be concerns with imprecision but not with inconsistency (no additional threshold is being crossed when switching from the fixed‐ to the random‐effects model). In the GRADE context – and if we only had this information ‐, we would be considering rating down for imprecision by two levels but not rating down for inconsistency. In their systematic review, Fraison et al. rated down one level for imprecision (considering only the number of participants), but not for inconsistency.

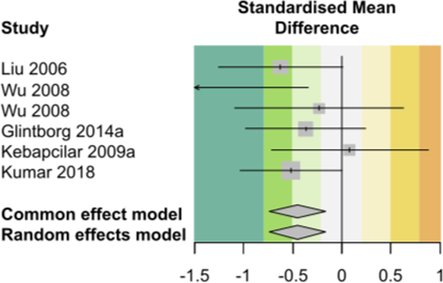

**Example for cluster III**

Zonneveld et al. have performed a systematic review assessing blood pressure drugs for preventing cardiovascular diseases in patients with a stroke or transient ischaemic attack [15]. One of the performed meta‐analysis involved comparing blood pressure lowering drugs *versus* placebo on occurrence of myocardial infarction. The meta‐analysis comprised 6 primary studies, including a total of 34,747 participants. The random‐effects absolute risk difference was of 2 fewer events per 1000 individuals (95%CI from 6 fewer events to 3 more events per 1000 individuals) [*I* ^2^ = 38.3%; DI = 0%; ASI = 0%]. Considering the decision thresholds used for mortality events (Table S1), neither the CI of the random effects meta‐analytical estimate or the fixed effects meta‐analytical estimate cross any decision threshold.

The fact that neither the CI of the fixed effects meta‐analytical estimate nor the CI of the random effects one cross any decision threshold indicates that there may not be concerns with either imprecision or inconsistency, respectively. In the GRADE context—and if we only had this information ‐, we would possibly not be considering rating down for either imprecision or inconsistency. In their systematic review, Zonneveld et al did not rate down for imprecision or inconsistency.

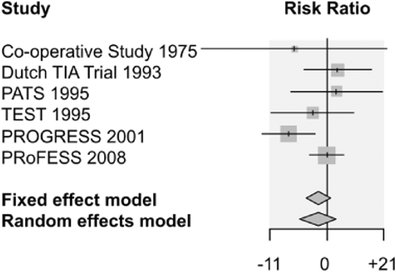

**Example for cluster IV**

Tao et al. have performed a systematic review assessing glutamine supplementation for critically ill adults [16]. One of the performed meta‐analysis compared glutamine supplemented‐nutrition to non‐supplemented nutrition on long‐term mortality. The meta‐analysis comprised 11 primary studies, including a total of 2277 participants. The random‐effects absolute risk difference was of 2 fewer events per 1000 individuals (95%CI from 61 fewer events to 37 more events per 1000 individuals) [*I* ^2^ = 30.2%; DI = 79.4%; ASI = 20.9%]. Considering the decision thresholds used for mortality events (Table S1), the CI of the random effects meta‐analytical estimate would cross five decision thresholds, while the CI of the fixed effects meta‐analytical estimate would cross four decision thresholds.

The fact that the CI of the fixed effects meta‐analytical estimate crosses four decision thresholds indicates that there may be concerns with imprecision. Moreover, the CI of the random‐effects meta‐analytical estimate crosses one additional decision threshold (with the prediction interval crossing even one more threshold), pointing to some concerns of inconsistency. In the GRADE context—and if we only had this information ‐, we would be considering rating down for imprecision by three levels and rating down for inconsistency by one level. In their systematic review, Tao et al did not rate down for imprecision or inconsistency.

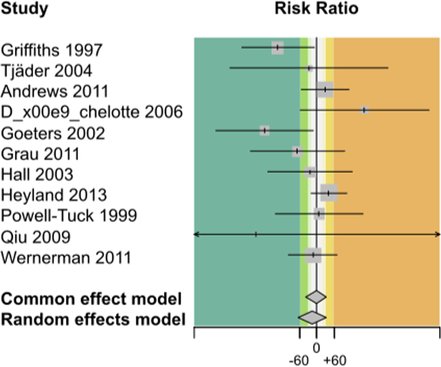

**Example for cluster V (and VI)**

Bighelli et al. have performed a systematic review assessing antidepressants and benzodiazepines for panic disorder in adult patients [17]. One meta‐analysis involved comparing tricyclic antidepressants *versus* benzodiazepines on the average frequency of panic attacks. The meta‐analysis included 6 primary studies with a total of 458 participants. The random‐effects SMD was 0.13 (95%CI = −0.24; 0.49) [*I* ^2^ = 66.8%; DI = 37.7%; ASI = 54.0%]. Considering the usual thresholds for SMDs, the random‐effects meta‐analytical estimate crosses two decision thresholds (trivial/small benefits and trivial/small harms). The fixed effects SMD would be of −0.01 (95%CI = −0.19; 0.18), with no decision threshold being crossed.

The absence of crossed decision thresholds by the fixed effects meta‐analytical estimate indicates that there are probably no serious concerns about imprecision. However, there are concerns with inconsistency, given that (i) the random‐effects meta‐analytical estimate crosses two decision thresholds, and (ii) all statistical measures of inconsistency display high values. In the GRADE context, if we only had this information, a rater may lower the certainty for inconsistency (if unexplained) by two levels, but not for imprecision. In their systematic review, Bighelli et a al did not evaluate the certainty of evidence for this outcome.

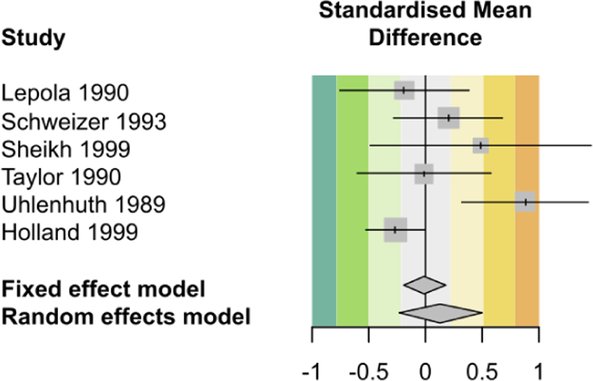

In brief:

- Clusters I–II (4.4% of the assessed meta‐analyses) were mostly associated with low inconsistency and high imprecision.
- Cluster III (48.1%) was associated with low inconsistency and low imprecision.
- Cluster IV (0.9%) was characterised by moderate to high inconsistency and high imprecision.
- Clusters V–VI (46.7%) were associated with high inconsistency, but low to moderate imprecision.

#### 3.1.2 Use of Different Metrics to Evaluate Inconsistency

##### 3.1.2.1 Hierarchical Cluster Analysis (Variables From Factor 1 Only)

We performed a second hierarchical cluster analysis, this time solely based on variables associated with inconsistency (‘factor 1 variables’) to identify potential “mismatches” between ∆DT, inconsistency indices (i.e., *I* ^2^, Decision Inconsistency Index [DI] [10], and Across‐Studies Inconsistency Index [ASI] [10]), and the information provided by PIs. We identified a total of six clusters (once again, we identified an optimal number of seven clusters, but one of the clusters had only three meta‐analyses and, therefore, we integrated it with the closest cluster). These clusters are summarised in Table S2. Overall, in clusters A, B, E, and F there was an overall alignment between ∆DT, inconsistency indices, and PIs. In clusters C and D, overall heterogeneity appears higher when considering inconsistency indices and PIs *vis‐a‐vis* ∆DT.

##### 3.1.2.2 Decision Tree Analysis

For clusters A, B, E and F, we assumed that the ∆DT were overall aligned with other inconsistency measures and reflect the number of levels to downgrade for inconsistency in GRADE up to a maximum of three levels (i.e., from high to very low). Considering the meta‐analyses that were part of those clusters, we built decision trees that aimed to predict the number of levels to downgrade. The best performing decision tree models were those that considered both (i) the difference in the number of DTs crossed by the PI and by the CI of the random‐effects meta‐analytical estimate, and (ii) inconsistency indices (Table S3). The decision tree with the highest accuracy and lowest complexity includes PIs and the *I* ^2^ (accuracy = 0.80 [95%CI = 0.78; 0.83]; *kappa* coefficient = 0.57) (Figure S2).

 The number of levels to downgrade for inconsistency by direct correspondence with ∆DT agreed 59.2% of the times with the number of levels to downgrade according to the decision tree rules (Figure S2). The lower the difference between the number of DTs crossed by the PI and the number of DTs crossed by the CI of the random‐effects meta‐analytical estimate, the higher the agreement with the number of levels to downgrade (Table S4): this agreement was particularly low if the difference in the number of crossed DTs was ≥ 4.

### 3.2 Proposed Approach to Disentangle Inconsistency and Imprecision and to Assess Inconsistency

**Figure 4:**
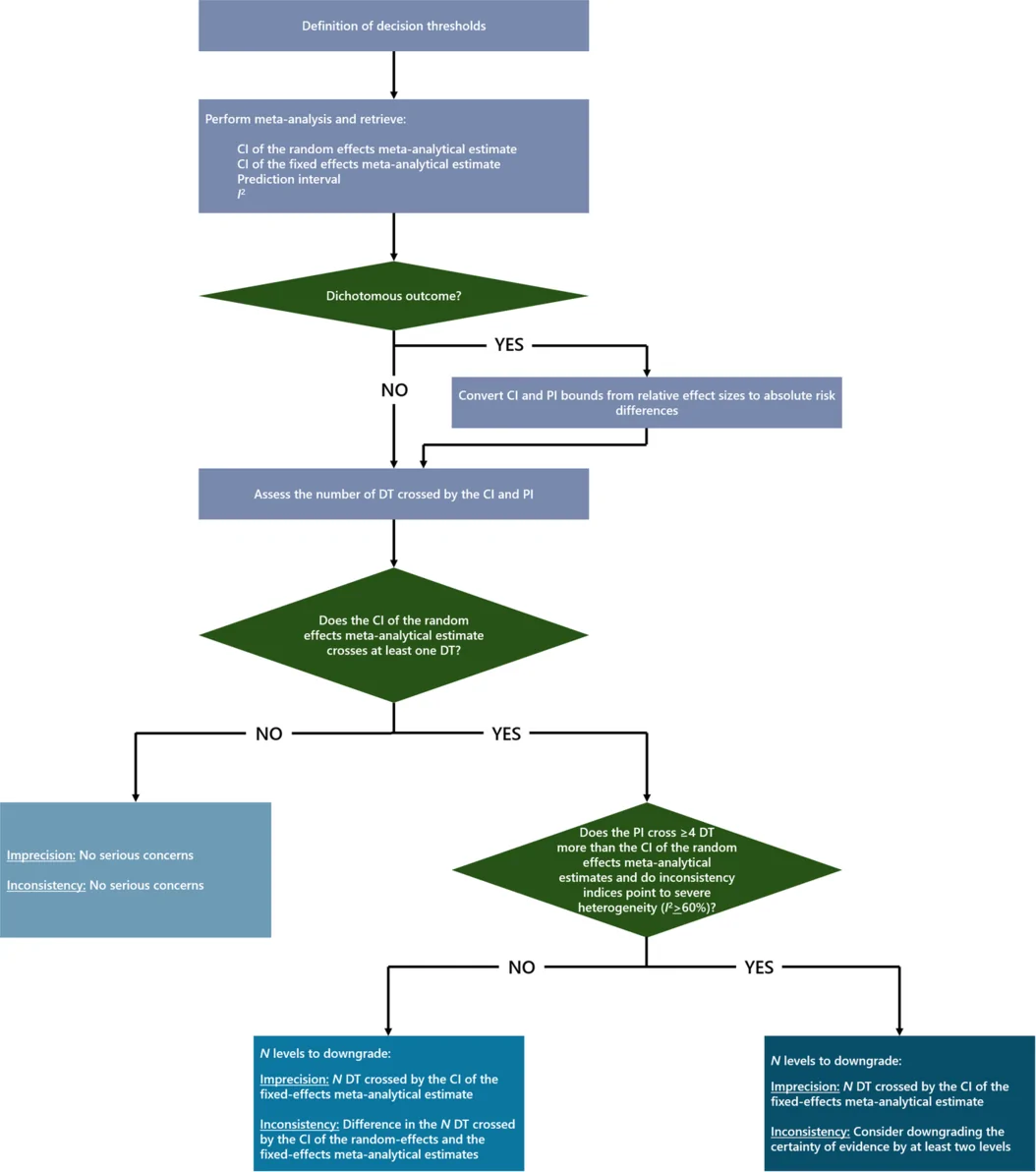
Suggested approach for disentangling inconsistency and imprecision. CI, Confidence interval; DT, Decision threshold; *N*, Number; PI, Prediction interval.

Based on these results, we propose an approach centered on the comparison of the number of DTs crossed by the CI of the random‐effects and the fixed‐effect meta‐analytical estimates as a starting point to disentangle inconsistency from imprecision (Figure 4). In brief, raters may start by assessing if the CI of the random‐effects meta‐analytical estimate crosses at least one DT. If no DTs are crossed by the CI of the random‐effects model, there are no serious concerns for both inconsistency and imprecision. If the CI cross DTs, the number of DTs crossed by the CI of the fixed‐effect meta‐analytical estimate indicate the ***degree of imprecision***. On the other hand, the difference between the number of DTs crossed by the CI of the random‐ *versus* the fixed‐effect model reflects ***the impact of inconsistency*** on the overall certainty. This is because the between‐study variability $\tau^{2}$ is included in the random‐effects but not in the fixed‐effect model.

After evaluating the fixed‐effects and the random‐effects CI, authors may evaluate PI. If the PI crosses at least four more DTs than the CI of the random‐effects meta‐analytical estimate, raters may evaluate if inconsistency is not being underestimated. In those situations, raters may consider the PI and inconsistency indices, such as the *I* ^2^, for example, considering the algorithm depicted in Figure S2. Of note, the consideration of a difference of at least four more DTs is grounded on the results of the comparison between the decision tree model and the difference in the number of DTs crossed by the random‐ and fixed‐effects meta‐analytical estimate.

Importantly, this approach to evaluating if the CIs of the fixed‐effects and the random‐effects meta‐analytical estimates cross DTs provides a starting point for judging imprecision and inconsistency. However, the final judgements should be based on a more comprehensive assessment.

## 4 Discussion

In this study, we analysed a sample of meta‐analyses published in Cochrane systematic reviews to discern the GRADE inconsistency and imprecision domains considering DTs. We propose that such disentangling can be achieved by a simple approach, involving the computation of the fixed‐ and of the random‐effects meta‐analytical estimates and assessment of their differences in the number of thresholds crossed by their CIs. PIs and inconsistency indices will be instrumental in some situations, particularly when the PI crosses at least four more DTs than the random‐effects CI.

### 4.1 Results Interpretation

The comparison of the number of DTs crossed by the CIs of the random‐effects and the fixed‐effect meta‐analytical estimates is a simple approach to disentangle inconsistency from imprecision. In fact, the precision of the fixed‐effect meta‐analytical estimate is not influenced by across‐study variability and, therefore, only reflects the imprecision domain. On the other hand, the width of the CI of the random‐effects meta‐analytical estimate is influenced by both within‐study and across‐study variability, reflecting both inconsistency and imprecision. Assessing the difference between the CIs of the random‐effects and the fixed‐effect meta‐analytical estimates may, therefore, provide relevant information for the inconsistency domain. In this study of a large sample of meta‐analyses, we tested several possibilities to compare the random‐effects and the fixed‐effect CI. Two of these possibilities involve computing the ratio of CI widths or the difference in the standard‐errors of the meta‐analytical measures but these metrics do not take into account DTs and do not have established cut‐off points. In addition, in our factor analysis they were not strongly related to other inconsistency metrics.

While we can establish a direct relation between the ∆DT and the number of levels to downgrade for inconsistency, there are situations in which this approach underestimates the degree of inconsistency. In particular, these are the situations where the PI crosses a much higher number of DTs (rule of the thumb considering the results of the decision tree analysis: at least four) compared to the CI of the random‐effects estimate. Such phenomenon is more commonly seen in meta‐analyses including a larger number of primary studies, where PIs and CIs display larger differences [18, 19], in part because CI are capable of accommodating more across‐study variability within narrower CIs.

For the assessment of inconsistency, emphasis on statistical measures commonly used to evaluate heterogeneity has limitations. The *I* ^2^ tends to yield biased results when used in meta‐analyses with few studies and to overestimate heterogeneity in meta‐analyses of primary studies with precise estimates [3, 8, 9]. In addition, it does not take into account DTs and information sizes. In fact, for a same high value of the *I* ^2^ statistic, inconsistency can be considered more serious if primary studies point to different effect size categories than if they all point to the same effect size categories (despite quantitative differences in their effect size measures resulting in large statistical heterogeneity). In addition, our results suggest against solely relying on the *p*‐value of the Cochran Q‐test to assess inconsistency. In our factor analysis, the *p*‐value of that test was not included in the factor that comprised the variables assessing inconsistency (factor 1). Finally, while PIs help evaluate inconsistency, they often spread through several DTs. Establishing a direct correspondence between the DTs crossed by PIs and the number of levels to downgrade due to inconsistency would therefore likely result in over‐penalising the inconsistency domain. Such a phenomenon may not be so evident if levels other than 95% were used for PI, but 95% PI continues to represent the standard and most frequently reported choice in the literature [19].

### 4.2 Strengths and Limitations

This study has important strengths. Despite recent studies suggesting a combined evaluation of imprecision and inconsistency [19], identifying whether the certainty of evidence (CoE) primarily results from inconsistency or imprecision can be particularly relevant, even if the final CoE ends up being the same. Firstly, this distinction allows for more transparency: when rating the CoE, users should provide a justification whenever there is a downgrade. Distinguishing between inconsistency and imprecision will allow for more precise justifications about downgrading, not only in terms of the affected domain but also regarding why the domain was rated down. In addition, if a concern with inconsistency is identified, users can explore whether there may be variables potentially explaining inconsistency: identification of such variables may preclude downgrading for inconsistency (the GRADE working group only recommends rating down the CoE for unexplained inconsistency [9]). In addition, recommendations for future primary studies will be directly influenced by understanding the reasons for lower certainty (Schünemann et al. 2026. In preparation). Serious inconsistency suggests that primary studies differ with regards to methods or clinical features that, in turn, require an identification of the variables explaining those differences (e.g., by subgroup analysis or meta‐regression). In such situations, recommendations for future research may include the suggestion that subsequent primary studies follow a more standardised methodology, are conducted in more similar clinical conditions, and are more transparent in relation to underreported potential variables related to heterogeneity. On the other hand, serious imprecision indicates that the existing body of evidence probably relies on underpowered studies, with direct consequences to reach a desired review information size. Therefore, a separate analysis of imprecision and inconsistency can have implications in terms of research priorities. Moreover, in the context of network meta‐analysis, the starting point for assessing the certainty of indirect evidence is based on the certainty of direct evidence without considering imprecision [20, 21].

Using meta‐epidemiological information from more than 8000 meta‐analyses allowed exploration of our approach in a large number of examples. Finally, we applied unsupervised classification methodologies, namely factor analysis, hierarchical clustering methods and decision trees, that provided a relatively straightforward approach and showed consistent results to disentangle the two domains out of a large dataset with multiple variables. It should be noted, however, that these approaches are based on statistical methods, with the results requiring subsequent interpretation (e.g., cluster analysis identifies variables that are strongly correlated with each other, with interpretation being required for the underlying factors). That is, out methodological approaches, while providing supportive evidence, do not identify or define per se conceptual constructs of imprecision or inconsistency, with the latter not even having a true gold‐standard with which results can be compared. Considering these aspects helps putting into context the results of this study and understanding some of the methodological approaches as well as the need for result interpretation.

This study also has some limitations. Generlizability may be limited by the fact that we only assessed systematic reviews published in the CDSR, with at least five primary studies, and that reported continuous outcomes or dichotomous outcomes for which DTs had been reported. While in meta‐analyses with a very small amount of primary studies, the *τ* ^2^ estimation can be highly imprecise (with some authors even discouraging meta‐analysis in systematic reviews of less than five studies) [22], 58% of the meta‐analyses published in the CDSR indeed had less than five primary studies. We excluded meta‐analyses of dichotomous outcomes without reported DTs, as some of the variables tested (e.g., ASI, DI, or number of DTs crossed by the fixed‐effects meta‐analytical estimate) require the *a priori* definition of DTs. However, this does not limit the practical applicability of our approach, as there are empirical methods to determine DTs for dichotomous outcomes [5, 6]. While these methods for empirically determining DTs were not feasible to apply to our large study (as such would have implied gathering information on the disutilities associated with a very large set of outcomes), they can be easily applied by authors of a systematic review dealing with a limited number of outcomes. Overall, while these sample restrictions can have implications for the generalisability of our meta‐epidemiological results (e.g., on the amount of meta‐analyses with low inconsistency and high imprecision), they probably do no impact our conclusions in terms of proposed approaches to disentangle inconsistency from imprecision. Finally, we have recalculated all included meta‐analyses using the random‐effects model and applying the restricted maximum likelihood method for computation of the *τ* ^2^. We did not test, however, if our results were robust to other methods to estimate the *τ* ^2^, even though differences are not expected to be large. In addition, we did not test the results we would obtain using the Hartung‐Knapp adjustment [23, 24]. It is expected that changes in the cluster composition (e.g., in the frequency of meta‐analyses that are part of each cluster, or even in the generated clusters themselves) could have occurred if other methods to compute the *τ* ^2^ had been adopted or if the Hartung–Knapp adjustment had been used. On the other hand, we do not expect a relevant change in the results of the factor analysis or in our final proposed approach. In fact, given that our suggested approach is based on the difference between the number of thresholds crossed, we believe it would still apply to meta‐analyses performed with Hartung‐Knapp adjustment or with the *τ* ^2^ computed by methods other than the restricted maximum likelihood.

### 4.3 Implications

Our approach has implications for practice, providing a starting point approach for evaluating inconsistency and imprecision. Future studies should evaluate the added value of the proposed approach on reliability in judgements about imprecision and inconsistency should be evaluated. This information will be required in order to develop updated GRADE guidance for how to separately evaluate imprecision and inconsistency. The fact that the proposed approach can fit into a decision rule makes it particularly suitable for automation. The GRADE Working Group is developing GRADErater, a module for supporting automated assessment of GRADE CoE based on decision rules, which can easily incorporate the proposed approach [25].

## 5 Conclusion

In conclusion, inconsistency and imprecision are two domains of the GRADE CoE assessment whose independent assessment has been challenging. After analyzing a large sample of meta‐analysis published in systematic reviews of the CDSR, we found that identifying the domain of primary concern may be supported by the computation of the fixed‐effect and of the random‐effects meta‐analytical estimates and by the assessment of the differences in the number of DTs crossed by their CI. The assessment of inconsistency can then be further supported by integrating information from the PI and statistical measures of heterogeneity, particularly when there is a large difference in the number of DT crossed by the PI *vis‐à‐vis* the CI of the random‐effects CI. Our findings improve the understanding of the distinct concepts of imprecision and inconsistency and may contribute to future revisions of GRADE guidance of these domains.

## Author Contributions

**Bernardo Sousa‐Pinto:** conceptualization, methodology, investigation, formal analysis, data curation, writing – original draft. **Antonio Bognanni:** methodology, writing – review and editing. **Manuel Marques‐Cruz:** methodology, writing – review and editing. **Ignacio Neumann:** methodology, writing – review and editing. **Rafael José Vieira:** methodology, writing – review and editing. **Sara Gil‐Mata:** methodology, writing – review and editing. **Elie Akl:** writing – review and editing, conceptualization. **Joerg J. Meerpohl:** writing – review and editing, conceptualization. **Holger J. Schünemann:** conceptualization, methodology, writing – review and editing, supervision. **Waldemar Siemens:** methodology, investigation, formal analysis, writing – original draft.

## Funding

The authors have nothing to report.

## Ethics Statement

Not applicable. This is a meta‐research study and, as a result, did not require an ethics approval statement.

## Consent

The authors have nothing to report.

## Conflicts of Interest

The authors declare no conflicts of interest.

## Permission to Reproduce Material From Other Sources

Not applicable. This study does not reproduce material from other sources.

## Reporting Guideline

This article follows the guidelines for reporting meta‐epidemiological methodology research proposed by Murad and Wang (10.1136/ebmed‐2017‐110713).

## Supporting information


Supporting File

## Data Availability

The data that support the findings of this study are available from the corresponding author upon reasonable request.
