# Supplementary material for: Disentangling the GRADE Domains of Inconsistency and Imprecision: An Approach Based on a Meta‐Research Study
Source: Cochrane Evid Synth Methods. 2026 Aug 31;4(5):e70102. doi: 10.1002/cesm.70102 (PMC13533124; doi:10.1002/cesm.70102)
Supplement: Supplementary file 1 [file CESM-4-e70102-s001.docx]

**Supplementary Methods**

**Data sources and eligibility criteria**

We retrieved all systematic reviews published in the Cochrane Database of Systematic Reviews (CDSR) up until February 2024, with a valid Digital Object Identifier (DOI) and available and meta-analytical data file. From these, we assessed all meta-analyses (i) with at least five primary studies, (ii) which did not correspond to subgroup analyses, and which had provided their results (iii.a) as continuous outcomes allowing for computing meta-analytical results as standardised mean differences (SMD), or (iii.b) as dichotomous outcomes, with outcomes corresponding to some of those for which decision thresholds (DTs) have been established in previous guidelines (Supplementary Table 1). Diagnostic test accuracy and network meta-analyses were not considered.

**Retrieved variables**

Each included meta-analysis was recalculated using the fixed-effects model and the random-effects model (with use of the restricted maximum likelihood method for computation of the τ^2^). For continuous outcome variables, we performed meta-analysis of SMD; for categorical outcome variables, we performed meta-analysis of risk ratios (RR), odds ratios (OR) or risk differences (RD) (depending on the effect sizes based on which meta-analysis was originally performed in the respective primary studies; no meta-analyses of hazard ratios were identified). In line with GRADE guidance for assessing the CoE, RR and OR (and the lower and upper bounds of the corresponding confidence intervals [CI] and prediction intervals [PI]) were converted into absolute risk differences (ARD), using the following equations:

$$ARD=-1000\times BR\times(1-RR)$$

$$ARD=-1000\times BR-\left( \frac{BR\times OR}{1-BR+(BR\times OR)} \right)$$

With “BR” corresponding to the baseline risk, here assumed to correspond to the frequency of events in the comparison group.

We added a continuity correction to zero cell values when recalculating meta-analysis of dichotomous outcome variables.

From each meta-analysis, we retrieved the following metrics (Box 1): (i) *I*^2^, (ii) Q-Cochran test *p*-value, (iii) Decision Inconsistency index (DI), (iv) Across-Studies Inconsistency index (ASI), (v) number of DTs crossed by the CI of the meta-analytical estimate in the fixed effects model, (vi) number of DTs crossed by the CI of the meta-analytical estimate in the random effects model, (vii) width (range of values) of the CI of the meta-analytical estimate in the fixed effects model, (viii) width of the CI of the meta-analytical estimate in the random effects model, (ix) standard-error of the meta-analytical estimate in the fixed effects model, (x) standard-error of the meta-analytical estimate in the random effects model, (xi) number of DTs crossed by the PI, and (xii) expected proportion of primary studies in each effect size category according to the PI. In addition, we have also retrieved information on the number of included primary studies and on the number of participants.

To assess the DTs crossed by the meta-analytical estimates, we considered three DTs: (i) trivial (or no) *versus* small differences, (ii) small *versus* moderate differences, and (iii) moderate *versus* large differences. For continuous outcomes, classical Cohen’s effect sizes (0.2, 0.5 and 0.8, respectively defining small, moderate and large differences) were used as DTs. For dichotomous outcomes, DTs were applied as absolute effect size measures (i.e., number of events per 1000 participants), as displayed in Supplementary Table 1 along with the corresponding sources. Importantly, these DTs were not used in the original systematic reviews of the CDSR, which were mostly published before the concept of DT was introduced, let alone implemented in GRADE guidance. Therefore, we needed to identify guidelines in which, for a set of outcomes, DTs were established and used.

**Disentangling between imprecision and inconsistency**

We performed a factor analysis to identify factors of metrics; that is, sets of metrics more closely associated with each other (and potentially preferably pointing to inconsistency or imprecision). We performed factor analysis based on the following the variables after they have been scaled:

- *I*^2^;
- Q-Cochran test *p*-value;
- DI;
- ASI;
- Number of DTs crossed by the CI of the meta-analytical estimate in the fixed effects model;
- Difference in the number of DTs crossed by the CI of the meta-analytical estimate in the random effects model compared to the fixed effects model (${\Delta DT}_{CI_{RE},CI_{FE}}$);
- Ratio of the widths of the CI of the meta-analytical estimate in the random effects *versus* the fixed effects model;
- Standard-error of the meta-analytical estimate in the fixed effects model;
- Difference in the standard-errors of the meta-analytical estimate in the random effects model compared to the fixed effects model;
- Number of DTs crossed by the PI;
- Difference in the number of DTs crossed by the PI and the CI of the meta-analytical estimate in the random-effects model;
- Expected proportion of studies summary (EPSS): The methodology used to compute the DI was applied to the expected proportion of primary studies in each effect size category, so as to have a single index summarising the spread of such proportions throughout the different effect size categories (index ranging from 0 to 100, with higher values indicating higher inconsistency). In brief, for each meta-analysis we are applying the following formula, with $\frac{N_{j}}{N}$ corresponding to the expected proportion of studies in each effect size category:

$$EPSS=\left( 1-\left( \frac{\frac{1}{2}\sum_{j}^{7} \left| \frac{N_{j}}{N}-\frac{1}{7} \right|}{\frac{6}{7}} \right) \right)\times100$$

Since the study aimed at identifying common factors associated with the aforementioned variables, we performed common factor analysis with principal factoring analysis as the extraction method. The number of factors was selected based on the number of eigenvalues larger than 1. We considered a factor loading of 0.30. The Bartlett’s test was used to assess the adequacy of factor analysis, with a *p*<0.05 pointing to such adequacy.

In order to assess the robustness of the factor analysis results, we subsequently applied hierarchical clustering methods. We performed hierarchical clustering using complete linkage and Euclidean distance. We clustered meta-analyses based on the variables more closely related to inconsistency and imprecision (as indicated by the results of factor analysis). We hypothesised that different variables of a same factor would point in the same direction within each cluster. Variables had been scaled before clustering. The number of assessed clusters was selected based on the visual inspection of the dendrogram and the gap statistic.

**Use of different metrics to evaluate inconsistency**

In order to evaluate how inconsistency indices and PIs can complement an assessment of heterogeneity centered on ${\Delta DT}_{CI_{RE},CI_{FE}}$, we performed hierarchical cluster analysis followed by decision trees. In detail, we started by performing hierarchical cluster analysis solely based on metrics more closely related to inconsistency, as indicated by the results of the factorial analysis. We performed hierarchical clustering of scaled variables using complete linkage and Euclidean distance. The number of assessed clusters was selected based on the visual inspection of the dendrogram and the gap statistic.

For the meta-analyses that were part of clusters for which there was an alignment between ${\Delta DT}_{CI_{RE},CI_{FE}}$, inconsistency indices and PIs, we assumed that the ${\Delta DT}_{CI_{RE},CI_{FE}}$would correspond to the number of levels to downgrade due to inconsistency (up to a maximum of three levels). We randomly divided that set of meta-analyses into a train set (80% of the sample) and a test set (20% of the sample). In the train set, we applied decision tree-based models so that we could identify decision rules predicting the number of levels to downgrade for inconsistency. Tested independent variables included the difference in the number of DTs crossed by the PI and by the CI of the random-effects meta-analytical estimate (i) alone, (ii) alongside the *I*^2^, and (iii) alongside an average of inconsistency indices (*I*^2^, DI and ASI). For each model, we tested three complexity parameters (0.01, 0.025, and 0.05). We applied the models to the test set, registering the corresponding accuracy – by comparison with the number of levels to downgrade based on ${\Delta DT}_{CI_{RE},CI_{FE}}$ –, kappa coefficient, and number of internal and leaf nodes. We then compared the approaches in which the number of levels to downgrade for inconsistency (i) would directly correspond to ${\Delta DT}_{CI_{RE},CI_{FE}}$, or (ii) would be based on the decision tree identified as providing the best balance between accuracy and complexity.

**Software**

All analyses were performed using the software R (version 4.5.0.), with use of the packages cochrane, metafor, psych, factoextra, cluster, caret, rpart and rpart.plot.

**Supplementary Box 1. Interpretative description of each cluster**

**Clusters I-II** represented 4.4% of the assessed meta-analyses and were mostly associated with low inconsistency, but high imprecision. Regarding inconsistency, the median *I*^2^ was of 0%, the median ASI ranged between 0.9% and 2.1%, and the median DI between 20.9% and 35.3%. The same number of decision thresholds was crossed by 94.2% of the meta-analytical estimates calculated by the fixed and the random effects model. In 89% of the cases, the prediction interval crossed at most more than one threshold than the random-effects meta-analytical estimate. In relation to imprecision, 70.2% of fixed effects meta-analytical estimates crossed at least two decision thresholds.

**Cluster III** represented 48.1% of the assessed meta-analyses and was associated with both low inconsistency and low imprecision. Regarding variables associated with the inconsistency-related factor, the median *I*^2^ was of 23.4%, the median ASI of 10.9%, and the median DI of 17.1%. In addition, for most meta-analyses (81.2%), the same number of decision thresholds was crossed by the meta-analytical estimates calculated by the fixed and the random effects model. In 40% of the cases, the prediction interval and the random-effects meta-analytical estimate crossed the same number of thresholds. As for variables associated with the imprecision-related factor, in more than 90% cases, the fixed effects meta-analytical estimate crossed at most one decision threshold.

**Cluster IV** represented 0.9% of the assessed meta-analyses, being characterised by moderate to high inconsistency and high imprecision. The median statistical measures of inconsistency conveyed disparate information: the median *I*^2^ was of 0% and the median ASI was of 2.5%. However, the DI was of 55.1%, in more than one third of the cases the random-effects model crossed one decision threshold more than the fixed-effects model, and in more than half of the cases the prediction interval crossed one more threshold. As for imprecision, 97.3% of the fixed effects meta-analytical estimates crossed more than three decision thresholds.

**Clusters V-VI** represented 46.7% of the assessed meta-analyses and were associated with high inconsistency, but low to moderate imprecision. The two clusters mostly differed in the number of decision thresholds crossed by the prediction interval (in cluster VI, practically all prediction intervals crossed six decision thresholds, while in cluster F such percentage was of 45.7%). The imprecision of the fixed effects meta-analytical estimates was mostly low – in 88.4% cases, the fixed effects meta-analytical estimate crossed at most one decision threshold.

**Supplementary Table 1. Decision thresholds (DTs) per 1000 individuals used for dichotomous outcomes**

| **Outcome** | **DTs – DT1/DT2/DT3** | **Reference** |
| --- | --- | --- |
| Discontinuation of treatments due to adverse events | 104/205/397 | ^a^ |
| Intensive care unit hospitalisation | 25/50/96 | ^b^ |
| Intracranial haemorrhage | 18/35/68 | ^b^ |
| Limb amputation | 21/41/80 | ^b^ |
| Major bleeding | 23/46/89 | ^b^ |
| Mortality | 16/31/60 | ^b^ |
| Myocardial infarction | 23/44/86 | ^b^ |
| Serious adverse events | 39/77/149 | ^a^ |

DT1=DT distinguishing trivial versus small effects; DT2=DT distinguishing small versus moderate effects; DT3=DT distinguishing moderate versus large effects. ^a^ DTs presented by Morgano et al. J Clin Epidemiol. 2024:111639 and obtained in the context of the Multiple Sclerosis International Federation (MSIF) guideline on Multiple Sclerosis treatments; ^b^ DTs presented by Cuker et al. Blood Adv. 2022;17(6):4915-4923.

**Supplementary Table 2. Results of the** **hierarchical clustering approach based on inconsistency metrics**

|  | **Cluster A** | **Cluster B** | **Cluster C** | **Cluster D** | **Cluster E** | **Cluster F** |
| --- | --- | --- | --- | --- | --- | --- |
| *N* (%) meta-analyses | 3068 (35.3) | 1168 (13.4) | 1493 (17.2) | 1970 (22.6) | 904 (10.4) | 100 (1.1) |
| *I*^2^ – median (IQR) | 0 (25.9) | 38.6 (29.7) | 66.0 (23.1) | 83.6 (16.1) | 93.2 (8.6) | 98.9 (2.4) |
| DI – median (IQR) | 16.7 (15.8) | 35.0 (17.2) | 38.2 (11.9) | 53.3 (10.1) | 58.6 (14.6) | 55.4 (21.5) |
| ASI – median (IQR) | 0.9 (11.6) | 22.6 (12.8) | 34.5 (10.0) | 44.1 (8.7) | 51.5 (10.2) | 55.9 (16.1) |
| Difference of thresholds crossed by the CI of RE and FE meta-analytical estimates – *N* (%) |  |  |  |  |  |  |
| 0 | 2785 (90.8) | 690 (59.1) | 1013 (67.9) | 221 (11.2) | 0 | 1 (1.0) |
| 1 | 280 (9.1) | 394 (33.7) | 480 (32.2) | 1370 (69.5) | 0 | 1 (1.0) |
| 2 | 3 (0.1) | 80 (6.8) | 0 | 379 (19.2) | 619 (68.5) | 1 (1.0) |
| ≥3 | 0 | 4 (0.3) | 0 | 0 | 285 (31.5) | 97 (97.0) |
| *N* decision thresholds crossed by PI – *N* (%) |  |  |  |  |  |  |
| 0 | 619 (20.2) | 0 | 0 | 0 | 0 | 3 (3.0) |
| 1 | 1315 (42.9) | 0 | 0 | 0 | 0 | 0 |
| 2 | 898 (29.3) | 171 (14.6) | 79 (5.3) | 0 | 0 | 0 |
| 3 | 236 (7.7) | 484 (41.4) | 479 (32.1) | 1 (0.1) | 1 (0.1) | 0 |
| 4 | 0 | 379 (32.4) | 538 (36.0) | 239 (12.1) | 22 (2.4) | 0 |
| 5 | 0 | 89 (7.6) | 259 (17.3) | 632 (32.1) | 114 (12.6) | 0 |
| 6 | 0 | 45 (3.9) | 138 (9.2) | 1098 (55.7) | 767 (84.8) | 97 (97.0) |
| Difference of thresholds crossed by PI and the CI of the RE meta-analytical estimate – *N* (%) |  |  |  |  |  |  |
| 0 | 1821 (59.4) | 82 (7.0) | 0 | 0 | 3 (0.3) | 64 (64.0) |
| 1 | 872 (28.4) | 385 (33.0) | 72 (4.8) | 0 | 41 (4.5) | 21 (21.0) |
| 2 | 375 (12.2) | 642 (55.0) | 605 (40.5) | 133 (6.8) | 270 (29.9) | 15 (15.0) |
| 3 | 0 | 58 (5.0) | 485 (32.5) | 906 (46.0) | 470 (52.0) | 0 |
| 4 | 0 | 1 (0.1) | 206 (13.8) | 778 (39.5) | 120 (13.3) | 0 |
| 5 | 0 | 0 | 45 (3.0) | 151 (7.7) | 0 | 0 |
| 6 | 0 | 0 | 80 (5.4) | 2 (0.1) | 0 | 0 |
| EPSS – median (IQR) | 17.1 (15.7) | 42.1 (17.1) | 44.9 (11.8) | 61.4 (12.3) | 66.6 (15.5) | 41.1 (19.1) |

ASI: Across-studies inconsistency index; DI: Decision inconsistency index; EPSS: Expected proportion of studies summary; IQR: Interquartile range; RE: Random effects

Green indicates low values in inconsistency or imprecision metrics, yellow indicates low-intermediate values, orange indicates high-intermediate values, and red indicates high values.

**Supplementary Table 3. Accuracy and kappa coefficients of the different tested decision tree-based models aiming at predicting the number of levels to downgrade for inconsistency**

| **Variables in the decision tree** | **Accuracy (95%CI)** | **Kappa coefficient** | ***N* internal nodes (*N* leaf nodes)** |
| --- | --- | --- | --- |
| PI |  |  |  |
| CP=0.010 | 0.734 (0.706;0.761) | 0.333 | 1 (2) |
| CP=0.025 | 0.734 (0.706;0.761) | 0.333 | 1 (2) |
| CP=0.050 | 0.734 (0.706;0.761) | 0.333 | 1 (2) |
| PI + *I*^2^ |  |  |  |
| CP=0.010 | 0.810 (0.785;0.833) | 0.591 | 6 (7) |
| CP=0.025 | 0.802 (0.777;0.826) | 0.574 | 3 (4) |
| CP=0.050 | 0.802 (0.777;0.826) | 0.574 | 3 (4) |
| PI + average of *I*^2^, DI and ASI |  |  |  |
| CP=0.010 | 0.842 (0.819;0.864) | 0.674 | 4 (5) |
| CP=0.025 | 0.842 (0.819;0.864) | 0.674 | 4 (5) |
| CP=0.050 | 0.825 (0.801;0.847) | 0.639 | 3 (4) |

ASI=Across-studies inconsistency index; CI=Confidence interval; CP=Complexity parameter (lower values indicate higher model complexity); DI=Decision inconsistency index; PI= Difference in the number of thresholds crossed by the prediction interval and the random-effects meta-analytical estimate

**Supplementary Table 4. Agreement between approaches suggesting the number of levels to downgrade for inconsistency based on (i) direct correspondence with the difference in decision thresholds (DTs) crossed by the confidence intervals (CIs) of the random-effects and fixed-effects meta-analytical estimates, and (ii) the decision tree displaying the best accuracy-complexity balance**

| **Difference in the *N* of DTs crossed by the PI and the *N* of DTs crossed by the CI of the random-effects meta-analysis (*N*)** | **Agreement between the two approaches in the *N* of levels to downgrade for inconsistency (%)** |
| --- | --- |
| 0 (1970) | 93.1% |
| 1 (1391) | 76.7% |
| 2 (2040) | 58.6% |
| 3 (1919) | 41.9% |
| 4 (1105) | 22.6% |
| 5 (196) | 0.5% |
| 6 (82) | 0% |

**Supplementary Figure 1. Flow diagram illustrating the selection of meta-analyses**

**Supplementary Figure 2. Results of the decision tree displaying the highest accuracy at the lowest complexity**

CI=Confidence interval; DT=Decision thresholds; PI=Prediction interval
